# Supplementary material for: Anti-Staphylococcal Activity of the Auranofin Analogue Bearing Acetylcysteine in Place of the Thiosugar: An Experimental and Theoretical Investigation
Source: Molecules. 2022 Apr 16;27(8):2578. doi: 10.3390/molecules27082578 (PMC9032686; doi:10.3390/molecules27082578)
Supplement: Supplementary file 1 [file molecules-27-02578-s001.zip › molecules-1691988-supplementary.pdf]

Antimicrobial activity of the Auranofin analogue bearing acetylcysteine in place of thiosugar against *Staphylococcus* strains. An experimental and theoretical investigation

Lorenzo Chiaverini [1§], Alessandro Pratesi [2§], Damiano Cirri [2], Arianna Nardinocchi [3], Iogann Tolbatov [4*], Alessandro Marrone [5], Mariagrazia di Luca [3*], Tiziano Marzo [1*] and Diego La Mendola [1]

[1] Department of Pharmacy, University of Pisa. Via Bonanno Pisano, 6, 56126, Pisa, Italy; tiziano.marzo@unipi.it (TM); lorenzo.chiaverini@farm.unipi.it

[2] Department of Chemistry and Industrial Chemistry (DCCI), University of Pisa, Via G. Moruzzi, 13, 56124 Pisa, Italy; alessandro.pratesi@unipi.it; damiano.cirri@dcci.unipi.it

[3] Department of Biology, University of Pisa, Via San Zeno 35-39, 56100 Pisa, Italy; mariagrazia.diluca@unipi.it (MDL); a.nardinocchi@studenti.unipi.it

[4] Institute of Chemical Research of Catalonia (ICIQ), The Barcelona Institute of Science and Technology, 43007 Tarragona, Spain; tolbatov.i@gmail.com

[5] Dipartimento di Farmacia, Università degli Studi "G. D'Annunzio" Chieti-Pescara, Via dei Vestini, I-66100 Chieti, Italy; amarrone@unich.it

§ Equally contributed

* Correspondence: tolbatov.i@gmail.com (IT); mariagrazia.diluca@unipi.it (MDL); tiziano.marzo@unipi.it (TM).

Characterization of AF-AcCys

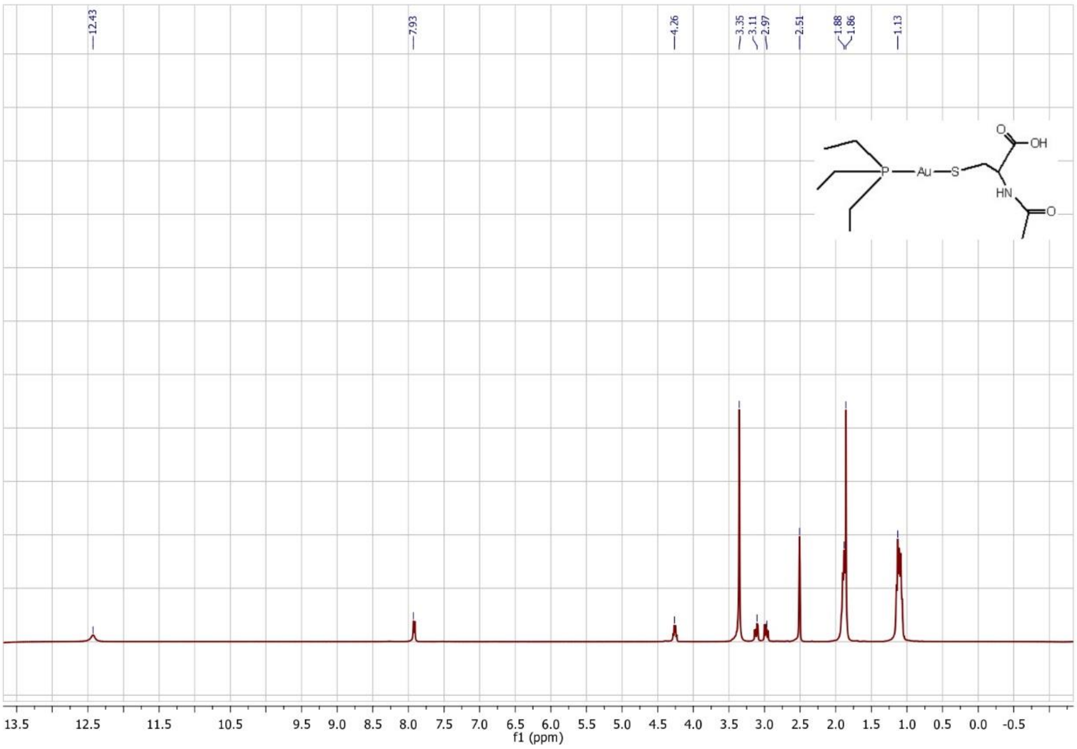

Figure S1: ^{1}HNMR (400 MHz; DMSO-d_6).

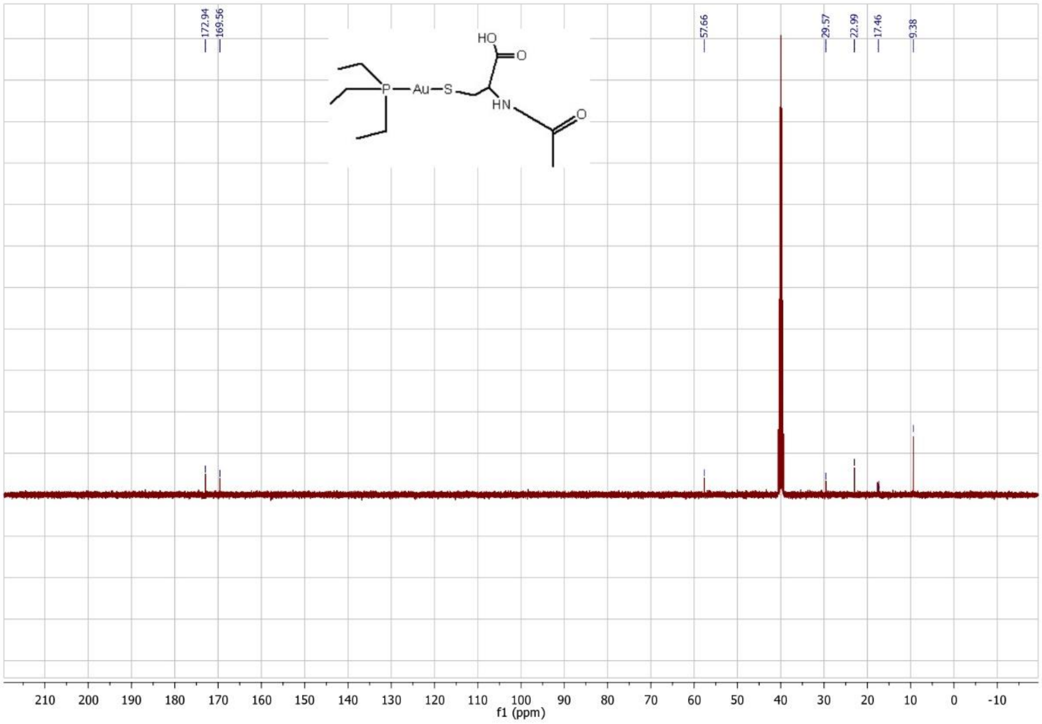

Figure S2: $^{13}C\{^{1}H\}$NMR (100 MHz; DMSO-d_6).

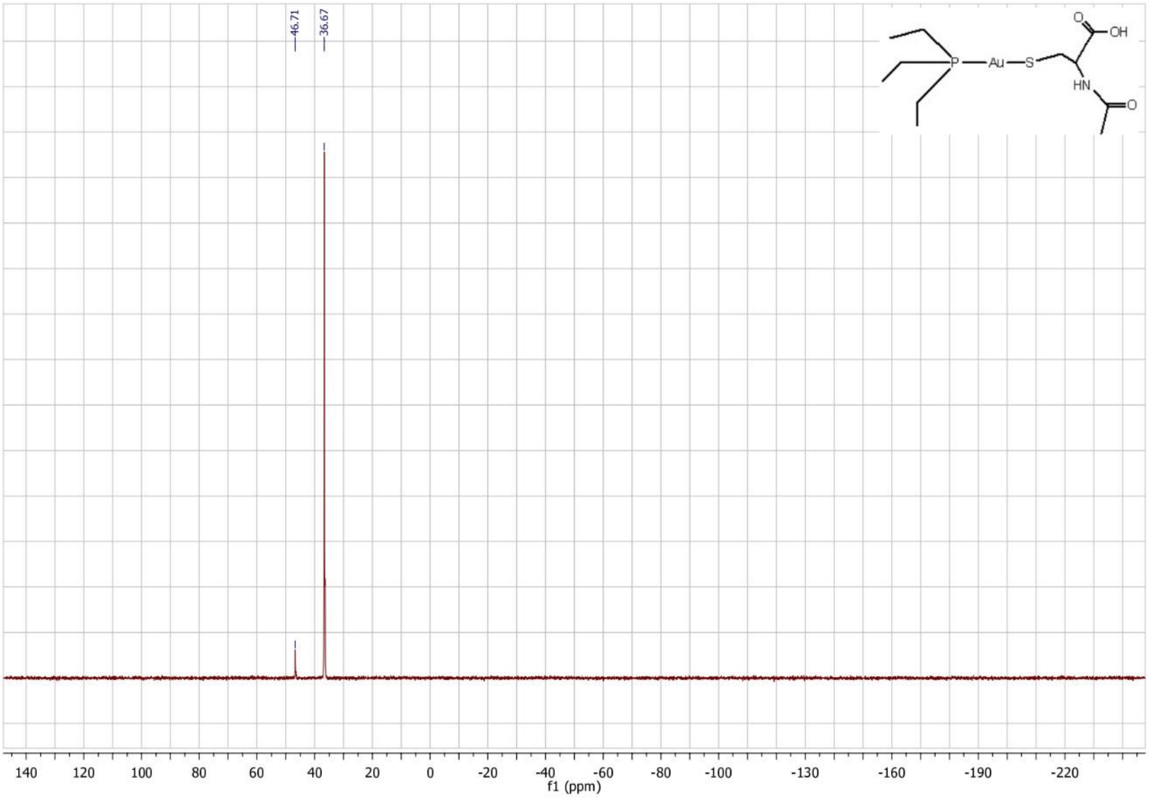

Figure S3: $^{31}P\{^{1}H\}$NMR (160 MHz; DMSO-d_6).

Stability studies of AF-AcCys and Auranofin

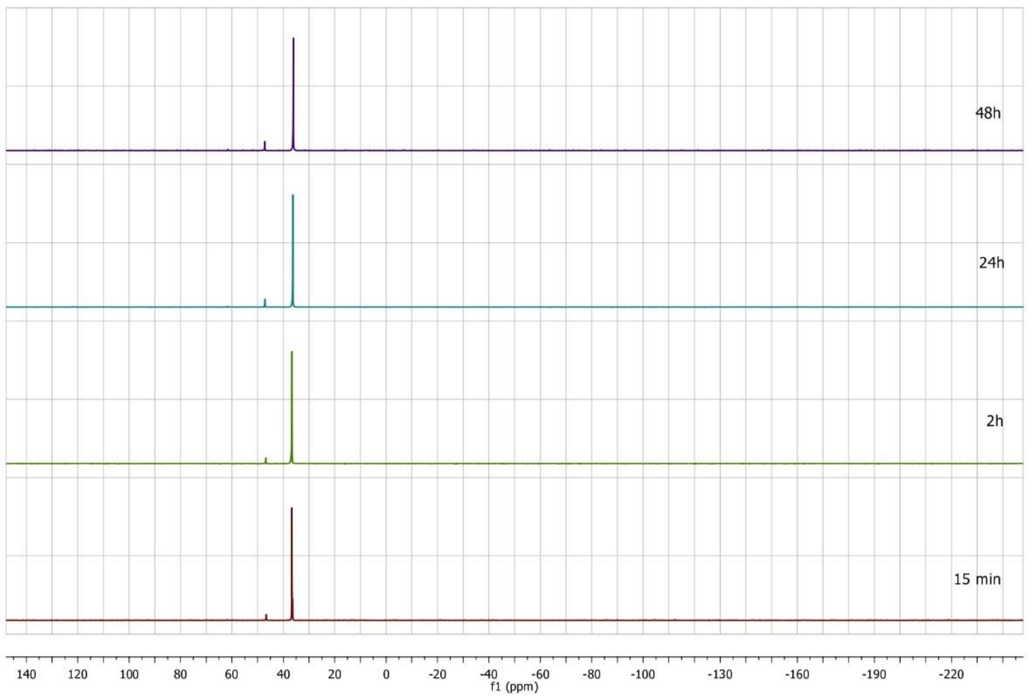

Figure S4: Stability study of AF-AcCys; $^{31}P\{^{1}H\}$NMR (160 MHz; DMSO-d_6/D_2O 1:1) δ = *46.48; 36.66.*

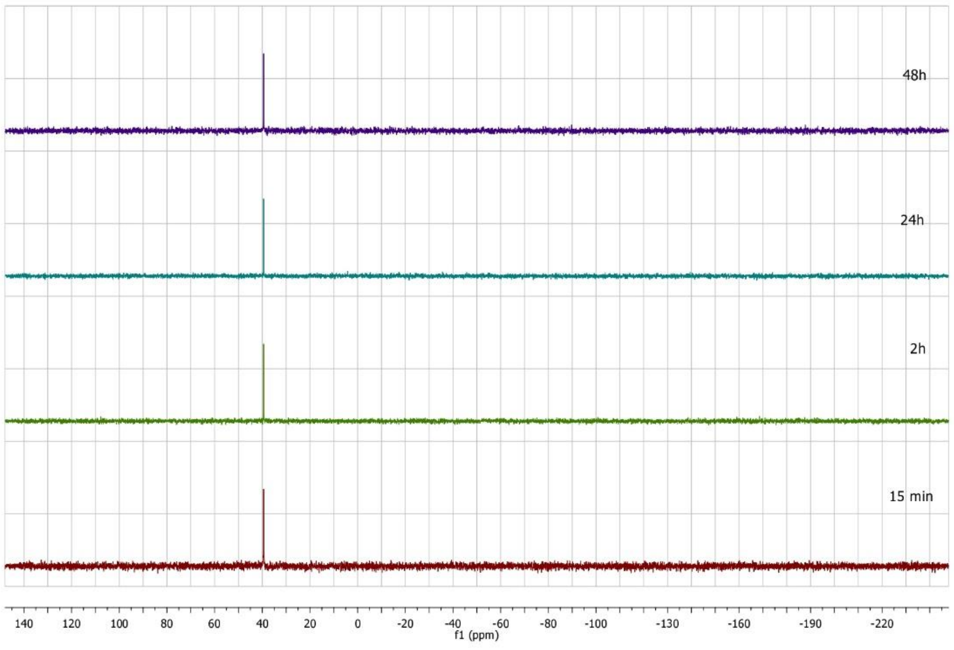

Figure S5: Stability study of Auranofin; $^{31}P\{^{1}H\}$NMR (160 MHz; DMSO-d_6/D_2O 2:1) δ = 39.45.

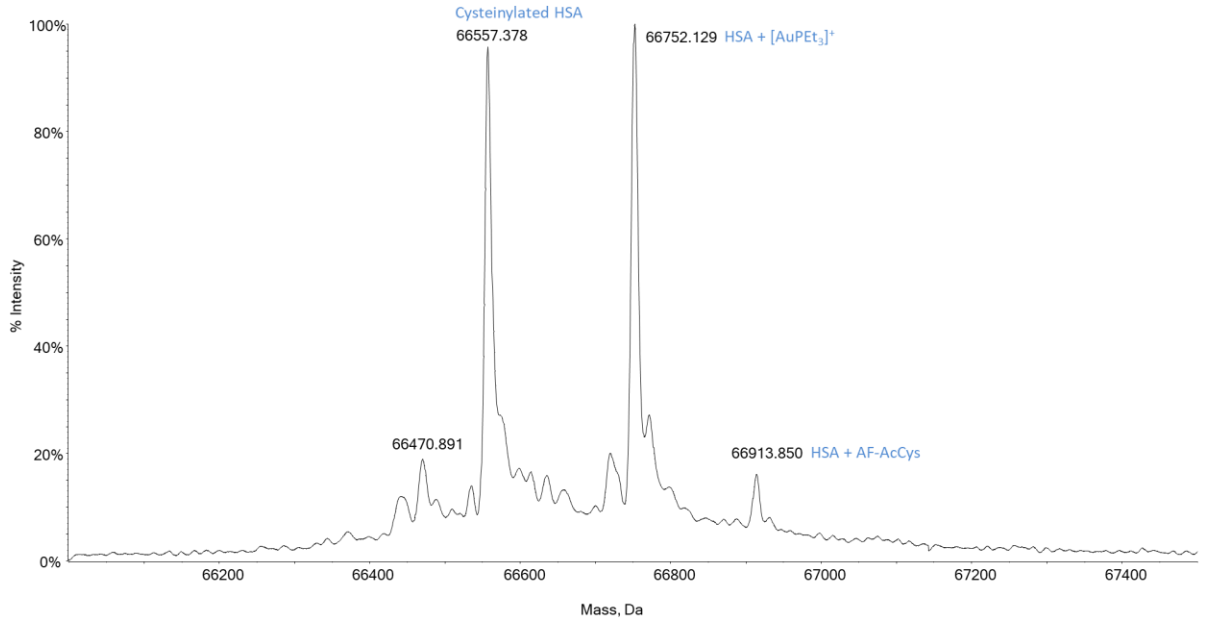

Figure S6. Deconvoluted ESI mass spectrum for HSA 5·10^{-6} M incubated with AF-AcCys (1:2 ratio) for 24 h at 37 °C in ammonium acetate solution 20 mM, pH=6.8.

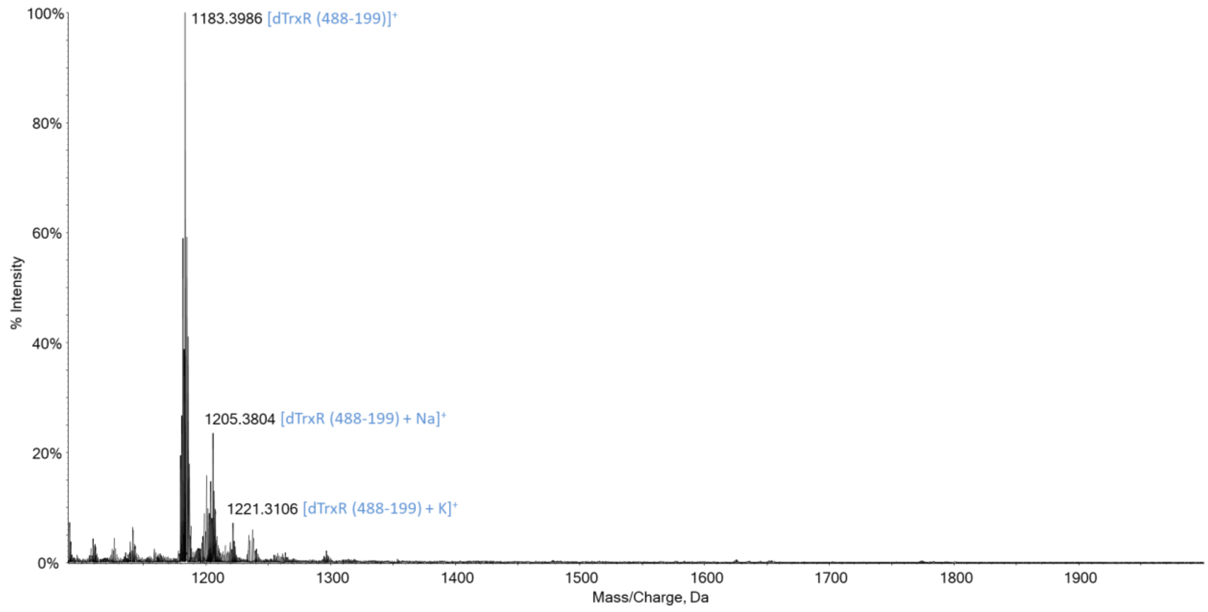

Figure S7. ESI mass spectrum of dTrxR(488–499) 10^{-6} M in water.

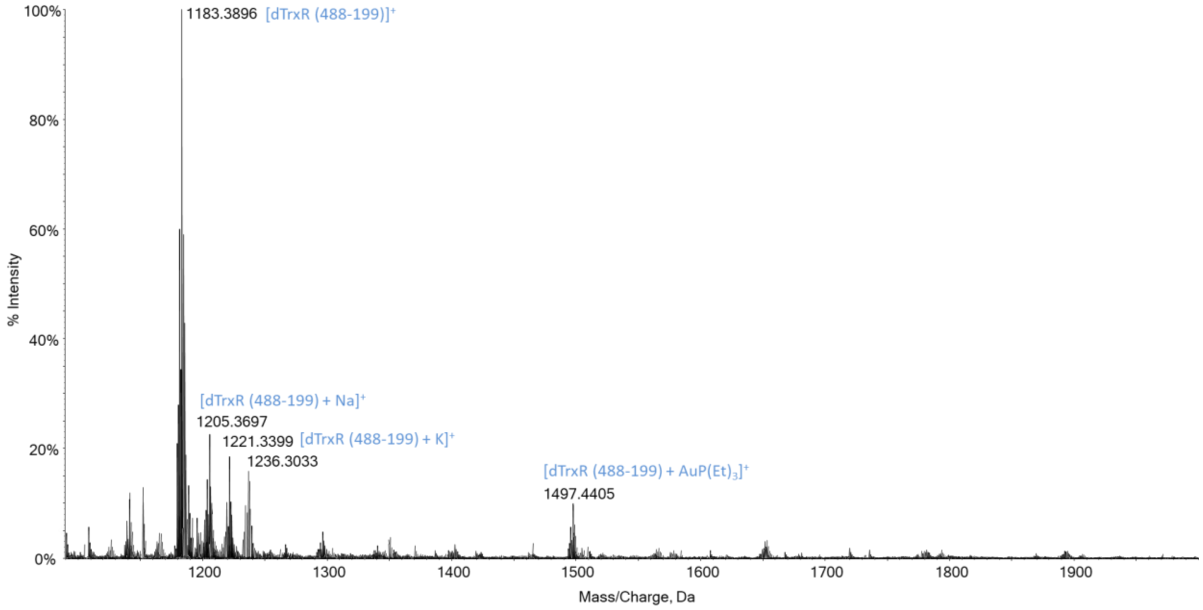

Figure S8. ESI mass spectrum of dTrxR(488–499) 10^{-6} M incubated with AF-AcCys (1:2 ratio) for 30 min at 37 °C in water.

Table S1. Mulliken charges distribution in AF and AF-AcCys. All values in a.u.

Fragment or atom	Mulliken charges	
	AF	AF-AcCys
Au	+0.14	+0.16
P	+0.17	+0.17
PEt_3	+0.37	+0.34
S	-0.51	-0.56
Thiosugar/AcCys	-0.51	-0.50
